# Pressure-induced superconductivity beyond magnetic quantum criticality in a Kondo ferromagnet

**DOI:** 10.1093/nsr/nwag119

**Published:** 2026-02-27

**Authors:** Yanan Zhang, Yongjun Zhang, Jiawen Zhang, Kaixin Ye, Dajun Su, Yanen Huang, Zhaoyang Shan, Jiyuan Li, Rui Li, Ye Chen, Xin Lu, Lin Jiao, Yu Liu, Michael Smidman, Frank Steglich, Huiqiu Yuan

**Affiliations:** New Cornerstone Science Laboratory, Center for Correlated Matter and School of Physics, Zhejiang University, Hangzhou 310058, China; Hubei Key Laboratory of Photoelectric Materials and Devices, School of Materials Science and Engineering, Hubei Normal University, Huangshi 435002, China; New Cornerstone Science Laboratory, Center for Correlated Matter and School of Physics, Zhejiang University, Hangzhou 310058, China; New Cornerstone Science Laboratory, Center for Correlated Matter and School of Physics, Zhejiang University, Hangzhou 310058, China; New Cornerstone Science Laboratory, Center for Correlated Matter and School of Physics, Zhejiang University, Hangzhou 310058, China; New Cornerstone Science Laboratory, Center for Correlated Matter and School of Physics, Zhejiang University, Hangzhou 310058, China; New Cornerstone Science Laboratory, Center for Correlated Matter and School of Physics, Zhejiang University, Hangzhou 310058, China; Hubei Key Laboratory of Photoelectric Materials and Devices, School of Materials Science and Engineering, Hubei Normal University, Huangshi 435002, China; New Cornerstone Science Laboratory, Center for Correlated Matter and School of Physics, Zhejiang University, Hangzhou 310058, China; New Cornerstone Science Laboratory, Center for Correlated Matter and School of Physics, Zhejiang University, Hangzhou 310058, China; New Cornerstone Science Laboratory, Center for Correlated Matter and School of Physics, Zhejiang University, Hangzhou 310058, China; New Cornerstone Science Laboratory, Center for Correlated Matter and School of Physics, Zhejiang University, Hangzhou 310058, China; New Cornerstone Science Laboratory, Center for Correlated Matter and School of Physics, Zhejiang University, Hangzhou 310058, China; New Cornerstone Science Laboratory, Center for Correlated Matter and School of Physics, Zhejiang University, Hangzhou 310058, China; New Cornerstone Science Laboratory, Center for Correlated Matter and School of Physics, Zhejiang University, Hangzhou 310058, China; Max Planck Institute for Chemical Physics of Solids (MPI CPfS), Dresden 01187, Germany; New Cornerstone Science Laboratory, Center for Correlated Matter and School of Physics, Zhejiang University, Hangzhou 310058, China; Institute of Fundamental and Transdisciplinary Research, Zhejiang University, Hangzhou 310058, China; Institute for Advanced Study in Physics, Zhejiang University, Hangzhou 310058, China; State Key Laboratory of Silicon and Advanced Semiconductor Materials, Zhejiang University, Hangzhou 310058, China; Collaborative Innovation Center of Advanced Microstructures, Nanjing 210093, China

**Keywords:** superconductivity, magnetic quantum criticality, heavy fermion, high pressure

## Abstract

Quantum phase transitions are an established setting for emergent phenomena driven by strong electronic correlations, including strange metals and unconventional superconductivity. These phenomena have been explored extensively in Kondo-lattice materials tuned to an antiferromagnetic quantum critical point (QCP), but superconductivity emerging near ferromagnetic quantum criticality has not yet been observed, and the conditions under which it occurs in proximity to ferromagnetism remain undetermined. Here, we report a new setting for superconductivity in the ferromagnetic Kondo-lattice material Ce$_5$CoGe$_2$, which has a ferromagnetic ground state at ambient pressure and evolves to antiferromagnetism under applied pressure. The antiferromagnetic transition is suppressed to a zero-temperature QCP, accompanied by strange-metal behavior. Superconductivity does not occur at the QCP, but instead appears at pressures beyond the magnetic instability. These findings suggest that Ce$_5$CoGe$_2$ represents a distinct class of correlated materials exhibiting a unique scenario for the emergence of superconductivity, likely associated with unconventional pairing mechanisms beyond spin fluctuations.

## Teaser

This work reports the discovery of a new superconductor Ce5CoGe2, which exhibits a novel scenario for superconductivity in correlated ferromagnets,whereby applying pressure to the Kondo ferromagnet Ce5CoGe2 first induces a change of magnetic ground state, with superconductivity appearing at higher pressures after magnetism is suppressed.

## INTRODUCTION

**Figure 1.**
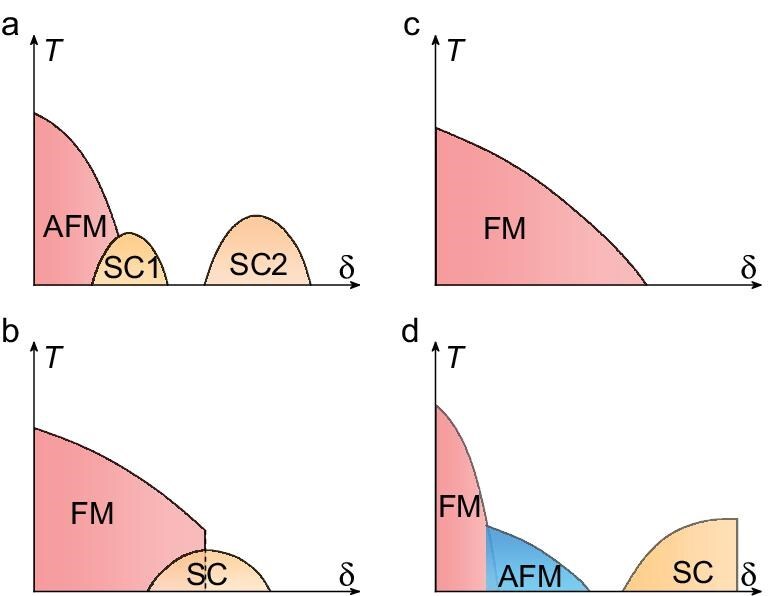
Schematic phase diagrams of superconductivity and magnetism in different quantum materials. (a) Representative phase diagram of CeCu$_2$Si$_2$, in which the first superconducting dome (SC1) emerges near the antiferromagnetic (AFM) quantum critical point (QCP). As the tuning parameter $\delta$ (pressure) increases, a second superconducting dome (SC2) appears, likely driven by valence fluctuations [14,17]. (b) Schematic phase diagram of uranium-based ferromagnetic superconductors, in which superconductivity (SC) coexists with ferromagnetic (FM) order. In these systems, the FM transition is typically first order, thereby avoiding a QCP [20]. (c) Schematic phase diagram of a continuous FM QCP, at which superconductivity has not yet been observed [23,25]. (d) Schematic phase diagram of Ce$_5$CoGe$_2$ under pressure. With increasing pressure, FM order first gives way to AFM order, which is subsequently continuously suppressed to zero temperature at an AFM QCP. Superconductivity emerges at higher pressures beyond the AFM QCP.

The close proximity of unconventional superconductivity to magnetism has been revealed in a variety of materials settings, including heavy-fermion superconductors [1], high-temperature cuprate [2], iron-based superconductors [3] and organic superconductors [4]. This has been interpreted within a framework in which superconductivity is driven by spin fluctuations [5,6]. This paradigm has been extensively investigated in heavy-fermion antiferromagnets [7–12], where the relative strengths of the competing Ruderman–Kittel–Kasuya–Yosida (RKKY) and Kondo interactions are tuned by experimentally applying non-thermal control parameters, and unconventional superconductivity often emerges upon the suppression of antiferromagnetic order at a quantum critical point (QCP). Moreover, in such systems superconductivity may also arise, driven by other degrees of freedom, such as quadrupolar [13] or valence fluctuations [14–16]. The latter has been proposed to explain the second superconducting state of pressurized CeCu$_2$Si$_2$, which is well separated from the antiferromagnetic (AFM) QCP [14,17,18], as illustrated in Fig. 1a.

In contrast, the evidence for superconductivity arising from ferromagnetic (FM) quantum criticality is more limited [19]. Although several uranium-based compounds exhibit the coexistence of superconductivity and FM order (Fig. 1b), in these cases the FM transition terminates abruptly under pressure in a first-order transition, thereby avoiding a QCP [20–22]. On the other hand, pressure and doping have been reported to induce continuous FM QCPs in CeRh$_6$Ge$_4$ [23,24] and YbNi$_4$(P$_{1-x}$As$_x)_2$ [25], respectively (Fig. 1c), but no superconductivity has yet been observed in these materials. Another scenario in which FM quantum criticality is avoided involves a change of ground state from FM order to a spin-density-wave (SDW) or other form of AFM ground state upon tuning with pressure or doping. Typical examples include the Kondo-lattice materials CeRu$_2$Ge$_2$ [26], CeAgSb$_2$ [27] and CeRuPO [28] as well as transition-metal compounds NbFe$_2$ [29] and LaCrGe$_3$ [30], in which superconductivity has also not yet been observed.

Here, we report a new superconductor Ce$_5$CoGe$_2$, which represents a novel setting for superconductivity in proximity to magnetism. At ambient pressure, Ce$_5$CoGe$_2$ is a Kondo-lattice compound showing the coexistence of ferromagnetism and cluster-glass behavior below the Curie temperature ($T_{\mathrm{\mathrm{\mathrm{C}}}}$) of 10.9 K (Fig. S1) [31]. The resulting temperature–pressure phase diagram is shown schematically in Fig. 1d, where, under pressure, the FM order first gives way to AFM order. With further increases in pressure, the AFM order is continuously suppressed and vanishes at an AFM QCP. Interestingly, superconductivity does not emerge immediately upon the suppression of antiferromagnetic order, but appears at higher pressures, separated from the AFM instability.

## RESULTS AND DISCUSSION

**Figure 2.**
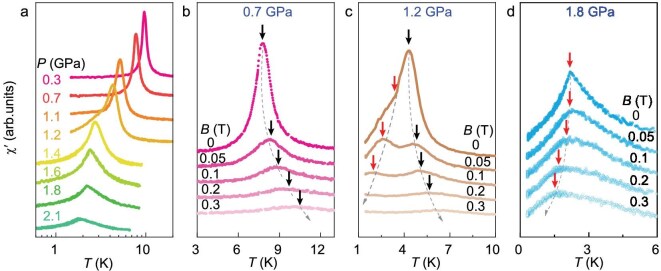
Pressure-induced FM-to-AFM transition in Ce$_{5}$CoGe$_{2}$. (a) Temperature dependence of the real part of the ac susceptibility, $\chi ^{\prime }(T)$, of Ce$_{5}$CoGe$_{2}$ under various pressures from 0.3 to 2.1 GPa. The $\chi ^{\prime }(T)$ data for Ce$_{5}$CoGe$_{2}$ are shown for (b) 0.7 GPa, (c) 1.2 GPa and (d) 1.8 GPa under various applied magnetic fields up to 0.3 T. The black and red arrows indicate the FM and AFM transitions, respectively. Note that the curves are vertically shifted for clarity.

In order to track the evolution of the magnetic ground state of Ce$_{5}$CoGe$_{2}$ with pressure, the temperature dependence of the ac magnetic susceptibility was measured under pressure, and the real part, $\chi ^{\prime }(T)$, is displayed in Fig. 2a under pressures up to 2.1 GPa. With increasing pressure, the peak in $\chi ^{\prime }(T)$ corresponding to the magnetic transition shifts from 9.7 K at ambient pressure to 1.7 K at 2.1 GPa, demonstrating a pressure-induced suppression of magnetic order. Meanwhile, measurements in applied magnetic fields (Fig. 2b–d) reveal distinct changes in the magnetic ground state under pressure. At lower pressures, such as 0.7 GPa, the transition moves to higher temperatures with increasing field, consistent with a ferromagnetic transition, whereas at 1.8 GPa the transition shifts to lower temperatures, which is characteristic of AFM order. At the intermediate pressure of 1.2 GPa, two peaks are observed with different field dependencies, indicating the coexistence of FM ($T_{\mathrm{\mathrm{\mathrm{C}}}} \approx 4.3$ K) and AFM ($T_{\mathrm{\mathrm{N}}} \approx 3.5$ K) transitions at this pressure. Furthermore, the frequency dependence of $\chi ^{\prime }(T)$ indicates that the spin-cluster-glass behavior present at ambient pressure [31] vanishes within the AFM phase (Fig. S2). These results suggest that, upon applying pressure to Ce$_{5}$CoGe$_{2}$, the magnetic ground state changes from FM to AFM. The nature of the coexistence of FM and AFM at 1.2 GPa is not yet determined; it could be that the FM-AFM boundary is first order, with coexisting macroscopic FM and AFM domains in its vicinity, or there could be a more microscopic coexistence, for example if the ordering on the four inequivalent Ce sites evolves differently with pressure.

**Figure 3.**
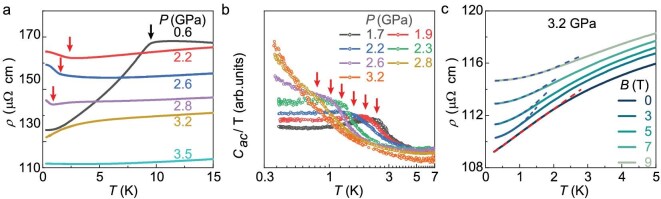
Quantum critical behavior in Ce$_{5}$CoGe$_{2}$. (a) Low-temperature dependence of the resistivity $\rho (T)$ of Ce$_{5}$CoGe$_{2}$ measured at pressures between 0.6 and 3.5 GPa. The red and black arrows indicate AFM and FM transitions, respectively. (b) Temperature dependence of the ac heat-capacity coefficient $C_{\rm ac}(T)/T$ of Ce$_{5}$CoGe$_{2}$ measured at pressures between 1.7 and 3.2 GPa, where the red arrows indicate AFM transitions. (c) The resistivity $\rho (T)$ under various applied magnetic fields at 3.2 GPa. The red dashed line marks the *T*-linear resistivity, corresponding to strange-metal behavior. The blue dashed lines show fits to a $T^{\rm 2}$ dependence, corresponding to Fermi-liquid behavior.

The electrical resistivity $\rho (T)$ of Ce$_{5}$CoGe$_{2}$ is displayed in Fig. 3a and Fig. S3. Ce$_{5}$CoGe$_{2}$ exhibits more metallic behavior as the pressure is increased from 0.6 to 5 GPa (Fig. S3a). The magnetic transition is also detected in the low-temperature $\rho (T)$ (Fig. 3a), where the FM transition appears as a kink below which $\rho (T)$ decreases rapidly, and $T_{\rm C}$ can be defined as the position of the maximum in the derivative ${\rm d}\rho (T)/{\rm d}T$ (Fig. S4). By contrast, there is an upturn in $\rho (T)$ upon cooling below the AFM transition, and the Néel temperature $T_{\rm N}$ corresponds to the minimum in ${\rm d}\rho (T)/{\rm d}T$ (Fig. S4), which could signal the opening of a gap [32]. Additional resistivity measurements in a piston–cylinder cell confirm the reproducibility of the resistivity upturn (Fig. S5). The upturn in $\rho (T)$ moves to lower temperatures with increasing pressure, which, together with the $\chi ^{\prime }(T)$ (Fig. 2a) and ac heat-capacity results (Fig. 3b and Fig. S6), demonstrates that the AFM transition is continuously suppressed to zero temperature, reaching a QCP at a critical pressure of $P_{\rm c}\approx 3.2$ GPa. At $P_{\rm c}$, $C_{\rm ac}(T)/T$ diverges, while the resistivity is linear in temperature from 2 K down to at least 250 mK (Fig. 3c), indicating strange-metal behavior at the QCP. When magnetic fields are applied along the *a* axis, $\rho (T)$ changes from a ${\sim } T$ to ${\sim } T^2$ dependence, suggesting the restoration of Fermi-liquid behavior in applied fields (Fig. S7).

**Figure 4.**
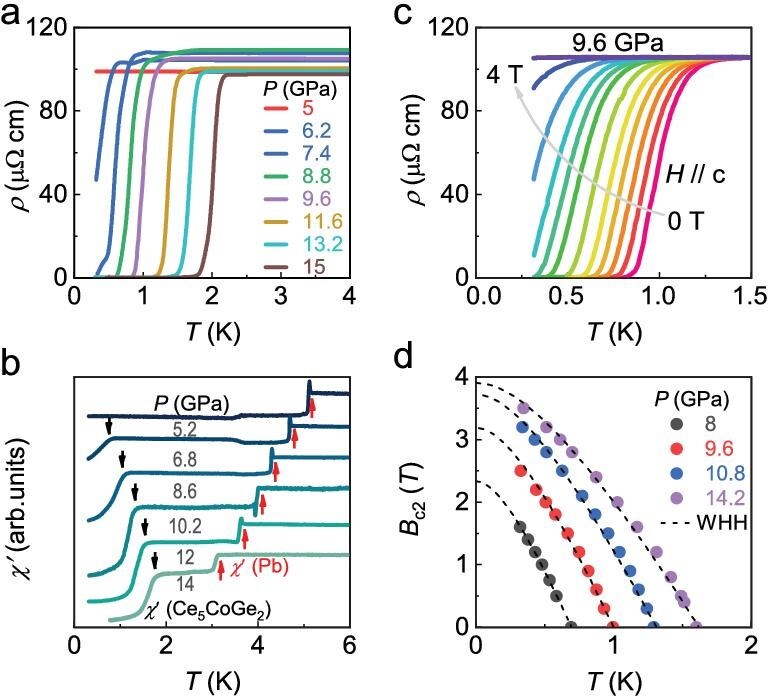
Superconductivity of Ce$_{5}$CoGe$_{2}$. (a) Low-temperature $\rho (T$) of Ce$_{5}$CoGe$_{2}$ measured between 5 and 15 GPa. (b) $\chi ^{\prime }(T)$ of Ce$_{5}$CoGe$_{2}$ at different pressures, where the superconducting transitions corresponding to the sample and to a Pb reference piece are indicated by black and red arrows, respectively. (c) The resistivity $\rho (T)$ under various applied magnetic fields at 9.6 GPa. (d) Temperature dependence of the upper critical field ($B_{\mathrm{c2}}$) at different pressures, with black dashed lines representing fits to the WHH model.

Figure 4a and Fig. S3b display $\rho (T)$ at higher pressures above $P_c$ up to 15 GPa. At 6.2 GPa, there is a sharp drop in $\rho (T)$ below 0.5 K, indicating the onset of superconductivity. At higher pressures, the transition temperature $T_{\rm sc}$ (determined from the temperature at which $\rho (T)$ drops by 50%) shifts to higher temperatures, reaching 2 K at 15 GPa, and zero resistance is realized at lower temperatures. To corroborate the occurrence of superconductivity, ac magnetic susceptibility measurements were performed under pressure (Fig. 4b), with a small piece of Pb in the pressure cell as a reference. The comparable jump in $\chi ^{\prime }(T)$ between Ce$_5$CoGe$_2$ and Pb provides clear evidence for a full shielding fraction in Ce$_5$CoGe$_2$ below the superconducting transition. Meanwhile, the normal-state resistivity remains metallic across the whole pressure range, but there is a slight upturn at low temperatures (Fig. S8), reminiscent of CeCu$_2$Si$_2$ under pressure [33].

**Figure 5.**
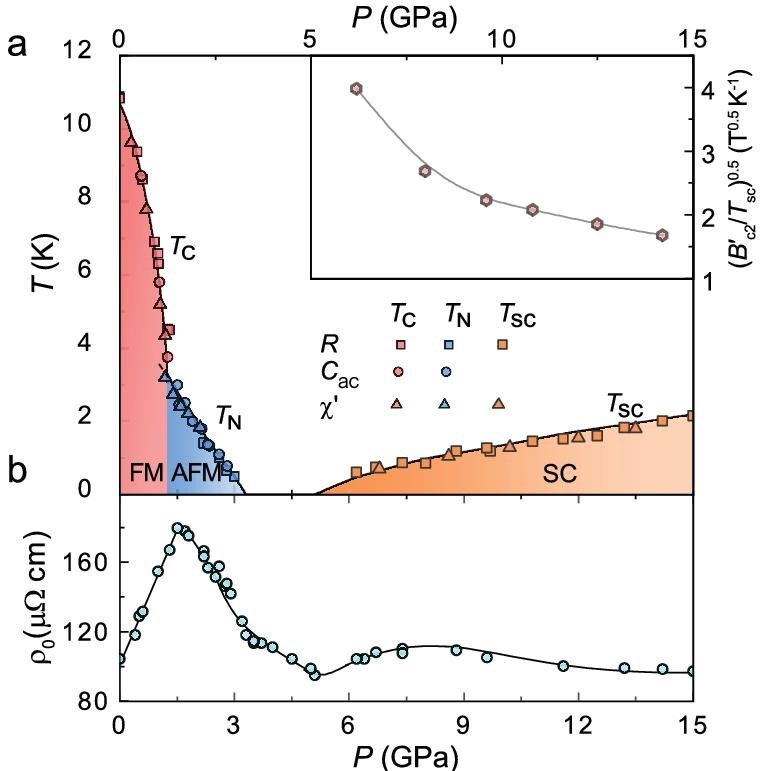
Phase diagram of Ce$_{5}$CoGe$_{2}$ under pressure. (a) Temperature–pressure phase diagram of Ce$_{5}$CoGe$_{2}$ based on resistivity, ac heat-capacity and ac susceptibility measurements. The pink, blue and orange symbols represent $T_{\rm C}$, $T_{\rm N}$ and $T_{\rm sc}$, respectively. The shaded regions correspond to the different labeled phases. The inset shows the pressure dependence of $(B^{\prime }_{\mathrm{c2}}/T_{\rm sc})^{0.5}$, which qualitatively describes the evolution of the effective carrier mass. (b) Pressure dependence of the low-temperature resistivity $\rho _{0}$, where $\rho _0$ is defined as the resistivity at 0.3 K. For pressures at which superconductivity occurs, $\rho _0$ corresponds to the normal-state resistivity just above the superconducting transition.

Figure 4c displays $\rho (T)$ at 9.6 GPa under different applied magnetic fields (see also Fig. S9 for other pressure points), while the derived upper critical fields as a function of temperature $B_{\mathrm{\mathrm{c2}}}(T)$ is shown in Fig. 4d; the data are well fitted using the Werthamer–Helfand–Hohenberg (WHH) model [34]. At all pressures, the upper critical field exceeds the weak-coupling Pauli limit ($B_{\mathrm{P}}$= 1.86 $T_{\rm sc}$). For example, at 8 GPa, $B_{\mathrm{P}} = 1.3\,\mathrm{T}$, yet the superconducting transition is still observed in applied fields above this value, and an extrapolated zero-temperature value of $B_{\mathrm{c2}}(0) = 2.3\,\mathrm{T}$ is obtained. The inset of Fig. 5a displays the pressure dependence of $(B^{\prime }_{\mathrm{c2}}/T_{\rm sc})^{0.5}$, where $B^{^{\prime }}_{\mathrm{c2}}$ = $-$(d$B_{\mathrm{c2}}$/d$T)_{T=T_{\rm sc}}$ is the initial slope of the upper critical field. Within the free-electron approximation, this ratio is proportional to the relative enhancement of the effective charge-carrier mass over the free-electron value, $m^{*}/m_0 \sim (B^{\prime }_{\mathrm{c2}}/T_{\rm sc})^{0.5}$ [35–37]. The value of $(B^{\prime }_{\mathrm{c2}}/T_{\rm sc})^{0.5}$, plotted versus $T_{\rm sc}$ for various heavy-fermion and other unconventional superconductors in Fig. S10, is comparable to that of canonical heavy-fermion compounds such as CeIn$_3$ [38] and CeCoIn$_5$ [39]. Upon further increasing the pressure, $(B^{\prime }_{\mathrm{c2}}/T_{\rm sc})^{0.5}$, and hence $m^{*}$, decreases, reaching a value close to that of the intermediate-valence superconductor PuCoIn$_5$ [15], as well as CeCu$_2$Si$_2$ in the high-pressure superconducting phase (Fig. S10) [40]. In addition, although $m^{*}/m_0$ is reduced by approximately a factor of two at $14.2\,\mathrm{GPa}$, it remains strongly enhanced compared with conventional BCS superconductors: $m^{*}/m_0$ in Ce$_{5}$CoGe$_{2}$ is still about 13.6 and 5.6 times larger than in MgB$_2$ and Nb$_3$Sn [41,42], respectively (Fig. S10). This comparison indicates that electronic correlations in Ce$_{5}$CoGe$_{2}$ are stronger than in typical conventional BCS superconductors.

The resulting temperature–pressure phase diagram of Ce$_5$CoGe$_2$ is shown in Fig. 5. The ground state evolves from FM order at ambient pressure to an AFM state above 1.2 GPa, and this antiferromagnetism is suppressed to a QCP at around $P_{\mathrm{c}}\approx 3.2$ GPa. Above the QCP at 6.2 GPa, superconductivity emerges, and $T_{\rm sc}$ increases monotonically with pressure up to at least 15 GPa. The pressure evolution of the low-temperature resistivity $\rho _{0}$ is shown in Fig. 5b, where there is a pronounced peak corresponding to the FM–AFM transition. Moreover, a broad hump in $\rho _0$ appears at elevated pressures following the emergence of superconductivity.

Overall these results demonstrate that Ce$_5$CoGe$_2$ manifests a new scenario for the interplay of magnetism and superconductivity, in which the superconductivity emerges in proximity to a QCP associated with the suppression of AFM order, which in turn replaces the ambient-pressure FM phase under pressure. While such a change of magnetic ground state occurs in several metallic ferromagnets [19], it is not typically associated with superconductivity. Moreover, unlike canonical antiferromagnetic heavy-fermion superconductors [7,37,43,44], the superconductivity does not appear to stem directly from a magnetic quantum critical point; instead, the superconducting dome is separate from the ordered phase (Fig. 1d), similar to the case of $\beta$-YbAlB$_4$ [16,45], suggesting that the superconductivity is not primarily driven by spin fluctuations.

Although the superconducting pairing state of Ce$_5$CoGe$_2$ remains to be explored in future studies, the Coulomb repulsion associated with such a strongly correlated underlying electronic state, as evidenced by the enhanced $m^{*}$, is unfavorable to conventional *s*-wave superconductivity with onsite pairing. These considerations raise the prospect that the superconductivity is driven by an alternative instability, such as valence fluctuations, which have been proposed to underlie the high-pressure superconducting dome of CeCu$_2$Si$_2$ [14,17,18]. An indication of the possible role of valence fluctuations in Ce$_5$CoGe$_2$ is the rapid reduction of the electronic effective mass upon increasing pressure (inset of Fig. 5a), which is characteristic of the crossover from a heavy-fermion to an intermediate-valence state [17]. In addition, valence fluctuations may enhance $\rho _{0}$, as seen in pressurized CeCu$_2$(Si$_{1-x}$Ge$_x)_2$ [18], and an increase in $\rho _{0}$ is also observed above 5 GPa in Ce$_5$CoGe$_2$, concomitant with the appearance of superconductivity. Moreover, a weak upturn in the resistivity with decreasing temperature, corresponding to a $-\log T$ dependence, is observed in the high-pressure superconducting regimes of both Ce$_5$CoGe$_2$ and CeCu$_2$Si$_2$ [33]; this feature is robust against magnetic fields (Fig. S8). Although its origin has not been determined, it may be attributed to unscreened magnetic moments in the mixed-valence regime. We note that in candidate valence-fluctuation-driven superconductors, $T_{\rm sc}$(*P*) can increase over a broad pressure range without an observed maximum. For example, it has been suggested that the superconductivity of PuCoGa$_5$, with a record $T_{\rm sc}$ of 18.5 K, is mediated by valence fluctuations [15], and, like Ce$_{5}$CoGe$_{2}$, the superconducting phase of PuCoGa$_5$ is robust, with $T_{\rm sc}$ changing only slightly over a wide pressure range [46]. It will therefore be important to extend the phase diagram to higher pressures in future measurements to establish the full evolution of the superconducting phase of Ce$_{5}$CoGe$_{2}$.

While the upper critical field exceeding the weak-coupling Pauli limit in a centrosymmetric superconductor could hint at a non-singlet pairing state, it should be noted that other effects—namely strong coupling and an effective Landé factor that deviates significantly from the free-electron value—may increase the paramagnetic limiting field. Therefore, it is of particular importance both to characterize the nature of the superconducting order parameter and pairing state, and to elucidate the dynamics associated with degrees of freedom such as spin and valence fluctuations that may drive superconductivity. Furthermore, it is necessary to understand the nature of the quantum criticality of Ce$_5$CoGe$_2$, where strange-metal behavior is observed only in a narrow pressure range close to $P_{\rm c}$. More broadly, this unique scenario for the interplay of ferromagnetism, antiferromagnetism and superconductivity, together with strange-metal quantum criticality, positions Ce$_5$CoGe$_2$ as a promising materials platform for exploring novel types of unconventional superconductivity in proximity to magnetic instabilities.

## METHODS

Single crystals of Ce$_5$CoGe$_2$ were grown using a self-flux method. Cerium ingot (Alfa Aesar, 99.9%), cobalt slug (Alfa Aesar, 99.95%) and germanium granules (PrMat, 99.9999%) were first arc-melted in a molar ratio of 9:3:1 under a titanium-gettered argon atmosphere. The obtained ingot was then placed in a tantalum crucible and sealed in an evacuated quartz tube. The tube was heated to $1150\, ^\circ {\rm C}$, held at this temperature for 24 h and then slowly cooled to $550\, ^\circ {\rm C}$.

Measurements under applied pressures up to 2.3 GPa were performed using a piston-cylinder-type pressure cell with Daphne 7373 as the pressure-transmitting medium. The applied pressure was determined from the shift in $T_{\rm sc}$ of a high-quality Pb single crystal [47]. Measurements at pressures up to 15 GPa were carried out in a diamond-anvil cell (DAC), where Daphne 7373 was used as the pressure-transmitting medium. The DAC was loaded together with several small ruby balls for pressure determination at room temperature using the ruby-fluorescence method [48]. Electrical-resistance measurements in both the piston-cylinder and diamond-anvil cells were performed using a standard four-probe method, with Au wires attached to the samples using silver conductive paste. Heat-capacity measurement under pressure were performed using an ac calorimetric technique, in which a heater attached to the sample generates a small temperature oscillation, $\Delta T$, by applying an ac current, and a chromel–AuFe (0.07%) thermocouple attached to the opposite side detects an ac voltage signal proportional to $\Delta T$. The ac magnetic susceptibility measurements in a piston-cylinder-type pressure cell were carried out using an in-house-designed coil system consisting of a drive coil, a pick-up coil and a compensation coil. The system was driven by an applied current of 0.1 mA at a frequency of 1523 Hz, and the voltage signal was detected using an SR-830 lock-in amplifier. Additional ac susceptibility measurements in a diamond-anvil cell were performed up to 14 GPa using a microcoil setup. The pick-up microcoil was wound from enameled Cu wire into a coil of radius $180\,\mu \mathrm{m}$ and positioned to maximize coupling to the sample in the sample chamber. A compensation coil was wound in the opposite direction outside the pick-up coil to reduce background signals. The drive coil was placed on the gasket and wound using a 45-$\mu \mathrm{m}$-diameter enameled wire with 250 turns. A small piece of lead (Pb), with a volume approximately one-third that of the sample, was included in the pressure cell as a reference. The resistance, ac magnetic susceptibility and ac heat capacity were measured using a Teslatron-PT system equipped with an Oxford $^3$He refrigerator and a Quantum Design Physical Property Measurement System (PPMS).

## Supplementary Material

nwag119_Supplemental_File
