# Supplementary material for: Pressure-induced superconductivity beyond magnetic quantum criticality in a Kondo ferromagnet
Source: Natl Sci Rev. 2026 Feb 27;13(9):nwag119. doi: 10.1093/nsr/nwag119 (PMC13185536; doi:10.1093/nsr/nwag119)
Supplement: nwag119_Supplemental_File [file nwag119_supplemental_file.docx]

**Supplementary Information for**

**Pressure-induced superconductivity beyond magnetic quantum criticality in a Kondo ferromagnet**

Yanan Zhang,^1^ Yongjun Zhang,^2^ Jiawen Zhang,^1^ Kaixin Ye,^1^ Dajun Su,^1^ Yanen Huang,^1^ Zhaoyang Shan,^1^ Jiyuan Li,^2^ Rui Li,^1^ Ye Chen,^1^ Xin Lu,^1^ Lin Jiao,^1^ Yu Liu,^1^ Michael Smidman,^1^ Frank Steglich,^1,3^ and Huiqiu Yuan,^1,4,5,6,7^

^1^ *Center for Correlated Matter and School of Physics, Zhejiang University, Hangzhou 310058, China*

^2^ *Hubei Key Laboratory of Photoelectric Materials and Devices, School of Materials Science and Engineering, Hubei Normal University, Huangshi 435002, China*

^3^ *Max Planck Institute for Chemical Physics of Solids (MPI CPfS), Dresden 01187, Germany*

^4^ *Institute of Fundamental and Transdisciplinary Research, Zhejiang University, Hangzhou 310058, China*

^5^ *Institute for Advanced Study in Physics, Zhejiang University, Hangzhou 310058, China*

^6^ *State Key Laboratory of Silicon and Advanced Semiconductor Materials, Zhejiang University, Hangzhou 310058, China*

^7^ *Collaborative Innovation Center of Advanced Microstructures, Nanjing 210093, China*

________________________

^*^ Correspondence: [liuyuccm@zju.edu.cn](mailto:liuyuccm@zju.edu.cn), [msmidman@zju.edu.cn](mailto:msmidman@zju.edu.cn), [hqyuan@zju.edu.cn](mailto:hqyuan@zju.edu.cn)


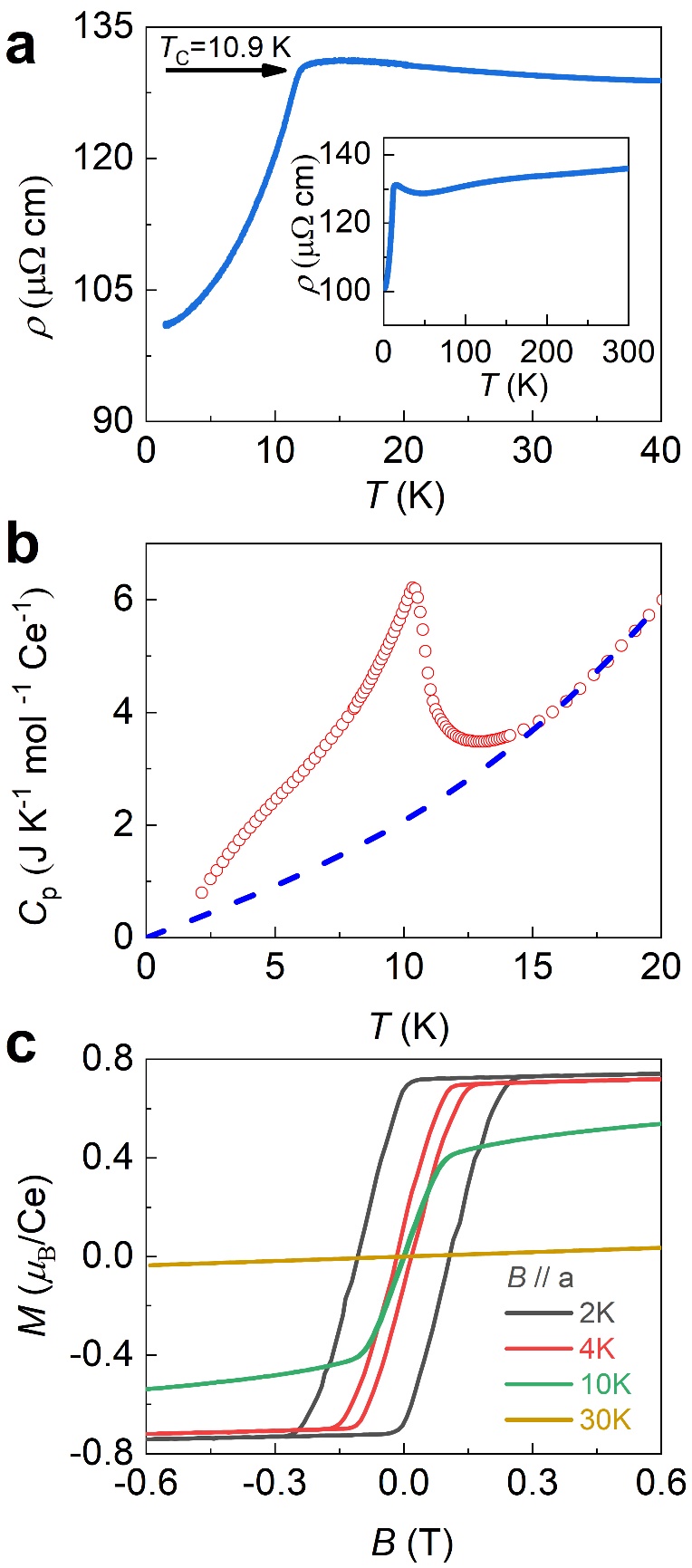


**Fig S1 | Electrical resistivity, magnetization, and specific heat** **of Ce_5_CoGe_2_ at ambient pressure.** **a,** Resistivity of Ce_5_CoGe_2_ at ambient pressure as function of temperature *ρ*(*T*) from 40 K down to 2 K, the black arrow indicates the ferromagnetic transition at *T*_C_ = 10.9 K. The inset presents the extended *ρ*(*T*) curve up to 300 K. **b,** Specific heat *C*_p_(*T*) of Ce_5_CoGe_2_ measured from 2 to 20 K, the blue dashed line represents fitting by *C*_p_(*T*) = γ*T*+β*T*^3^, which yields γ =180 mJ K^−2^mol^−1^Ce^−1^. **c,** Isothermal magnetization *M*(*H*) for fields applied along the *a* axis at selected temperatures.


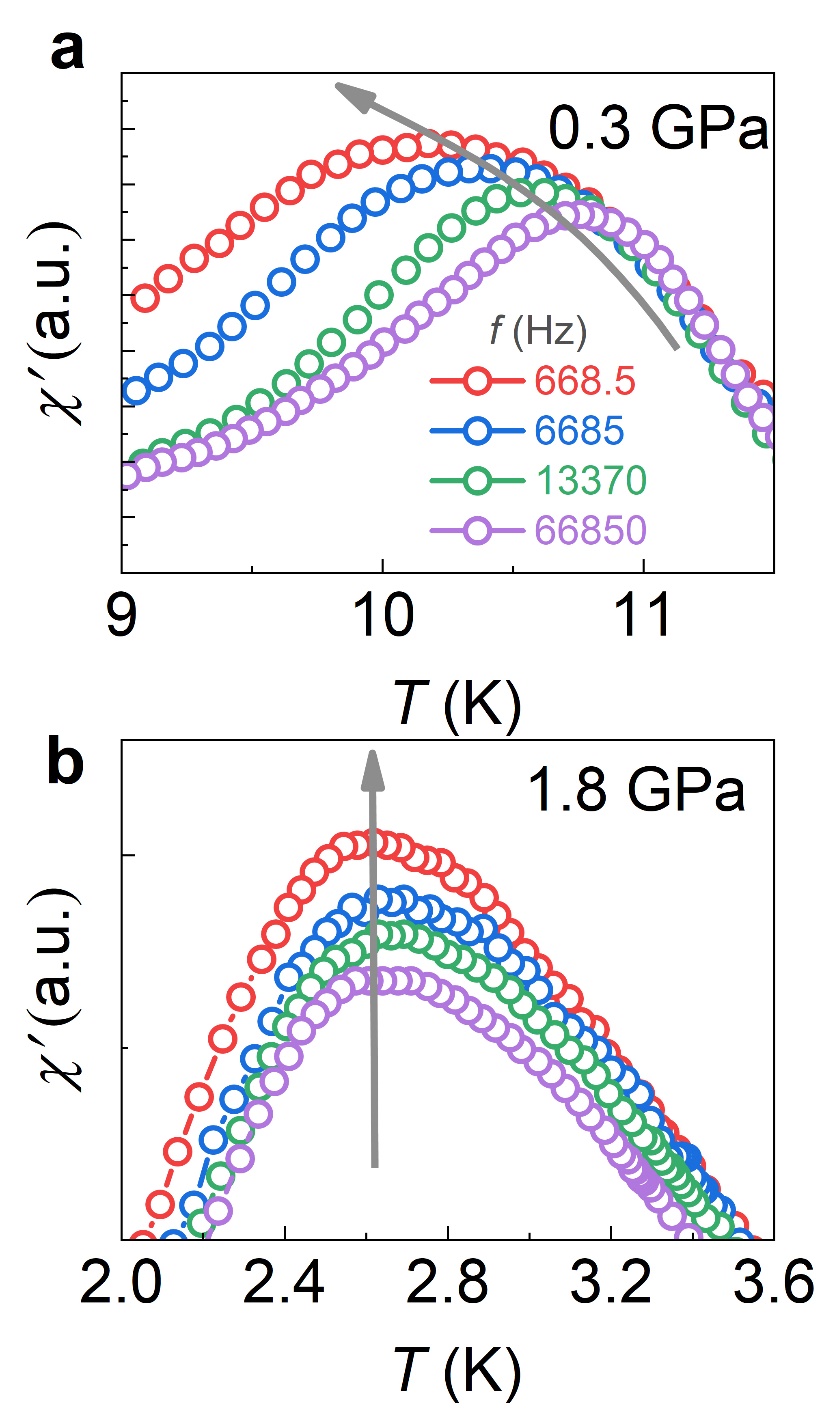


**Fig S2 | Temperature-dependent ac susceptibility χ′ of Ce_5_CoGe_2_ measured at different frequencies in the ferromagnetic (0.3 GPa) and antiferromagnetic (1.8 GPa) phases.** In the FM phase (panel **a**), the peak in χ′ shifts to lower temperatures as the frequency increases, suggesting frustration-induced spin-glass behavior ^[1]^. In contrast, the AFM phase (panel **b**) shows a transition temperature that remains essentially independent of frequency.


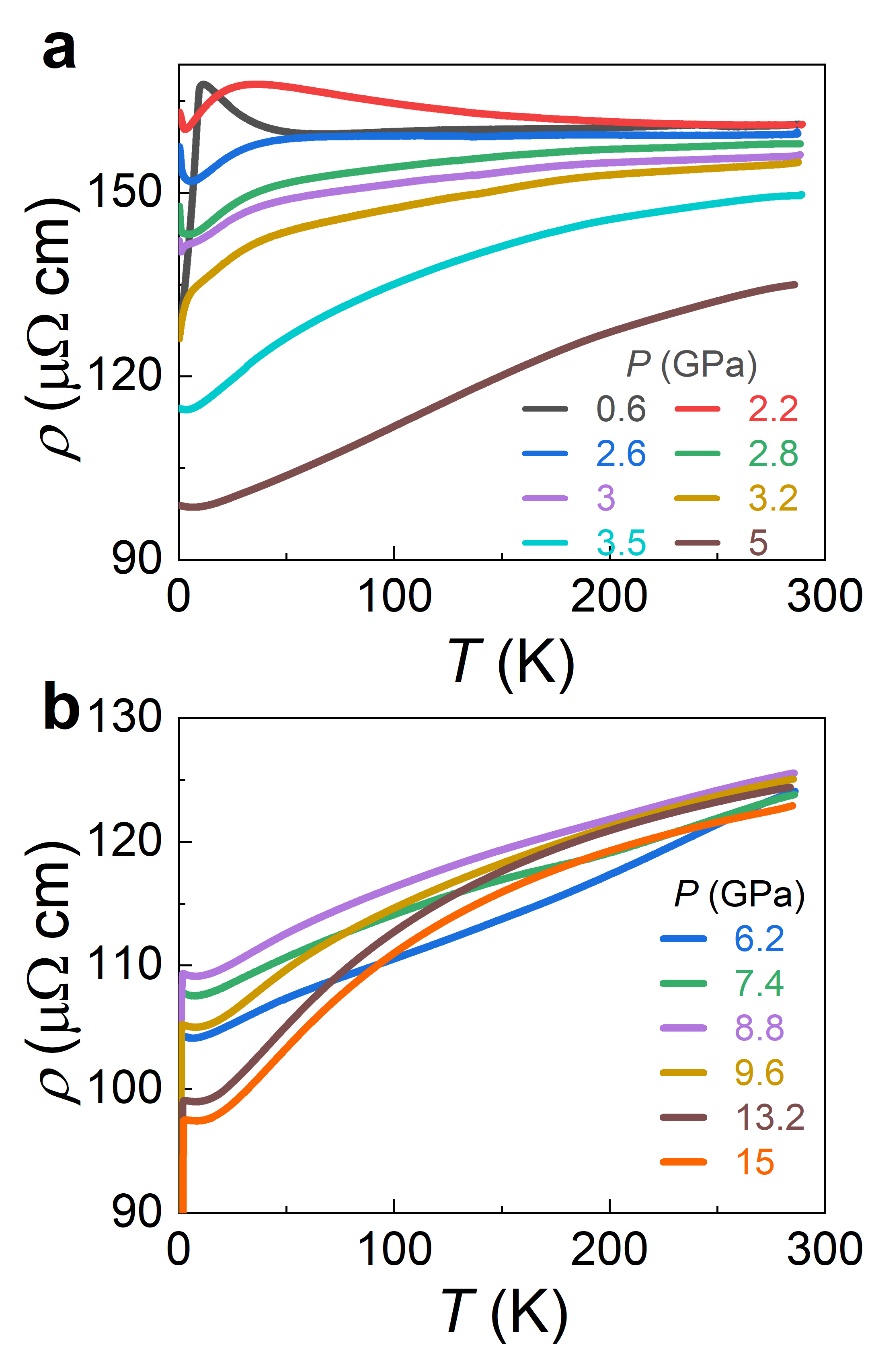


**Fig S3 |** **Temperature dependence of the resistivity *ρ*(*T*) of Ce_5_CoGe_2_ under various applied pressures.** *ρ*(*T*) is displayed under pressures of **a,** 0.6-5 GPa, **b,** 6.2-15 GPa. Data are shown over the temperature range 0.3 K to 270 K.


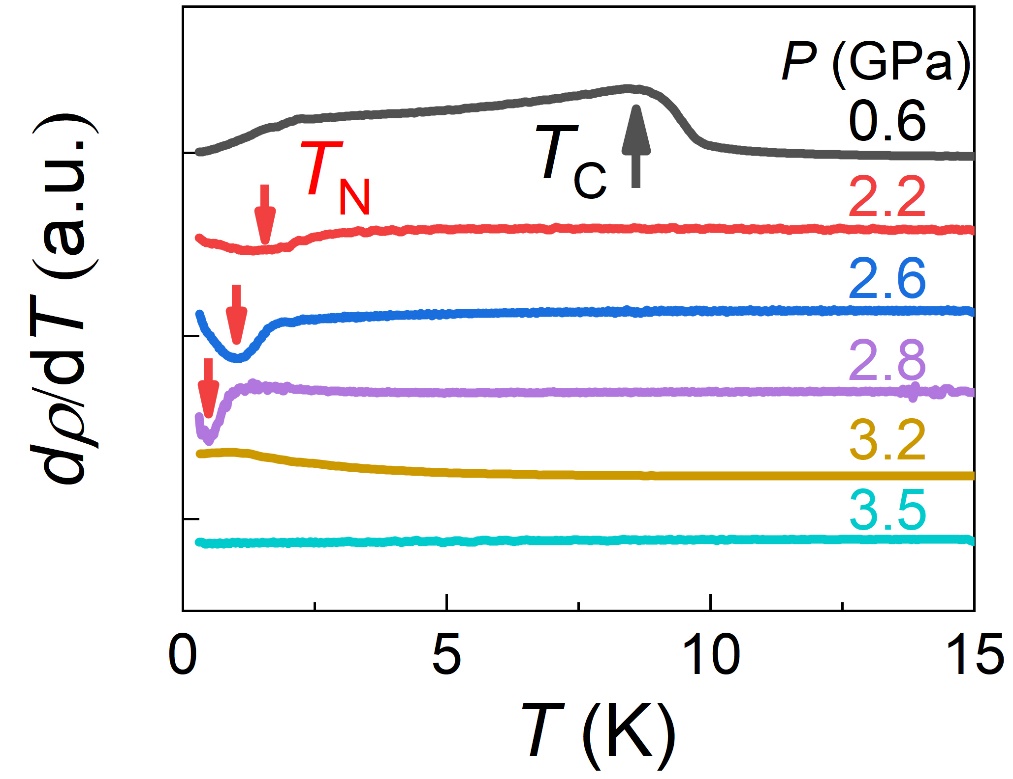


**Fig S4 | Temperature dependence of the derivative of the resistivity** **d*ρ*/d*T* under pressure.** The red arrows mark the antiferromagnetic transition temperature *T*_N_, while the black arrow indicates the ferromagnetic transition temperature *T*_C_. Note that the curves are vertically shifted for clarity.


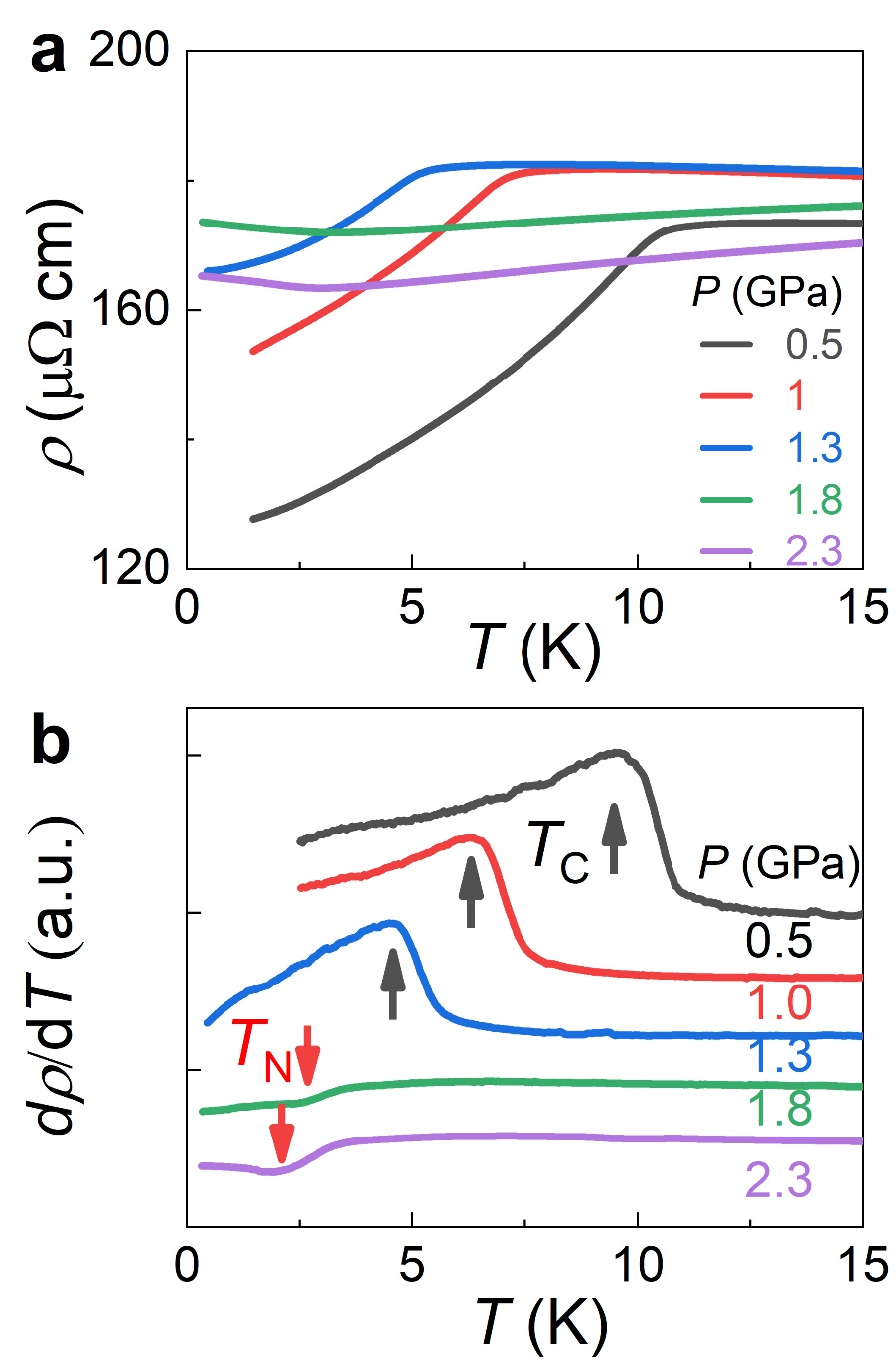


**Fig S5 | *ρ*(*T*)** **of Ce_5_CoGe_2_ measured under various pressures up to 2.3 GPa in a piston–cylinder cell. a,** The resistivity curves show that the ferromagnetic transition is gradually suppressed with increasing pressure up to 1.3 GPa. Above 1.3 GPa, there is an upturn in *ρ*(*T*) upon cooling below the AFM transition. **b,** Corresponding derivatives d*ρ*/d*T*, where the red arrows mark the antiferromagnetic transition temperature *T*_N_, while the black arrow indicates the ferromagnetic transition temperature *T*_C_.


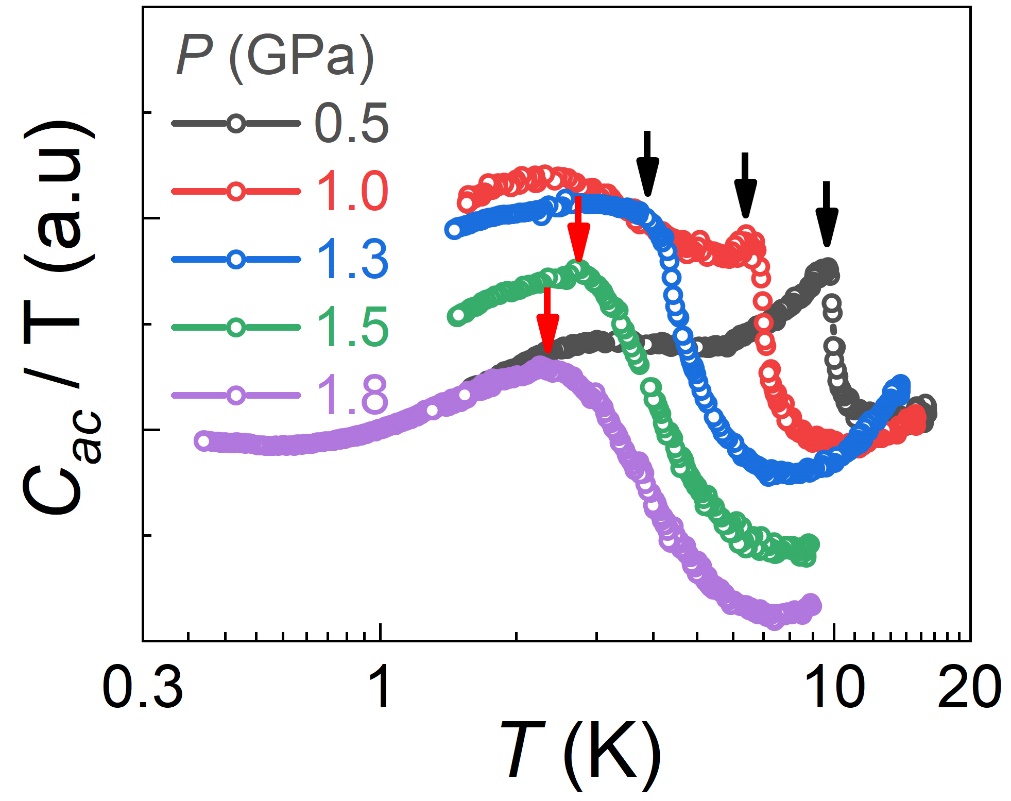


**Fig S6 | The ac heat capacity *C*_ac_/*T* of Ce_5_CoGe_2_ measured in a piston-cylinder cell at pressures ranging from 0.5 to 1.8 GPa.** The black and red arrows indicate the ferromagnetic and antiferromagnetic transitions, respectively. Note that the curves are vertically shifted for clarity.


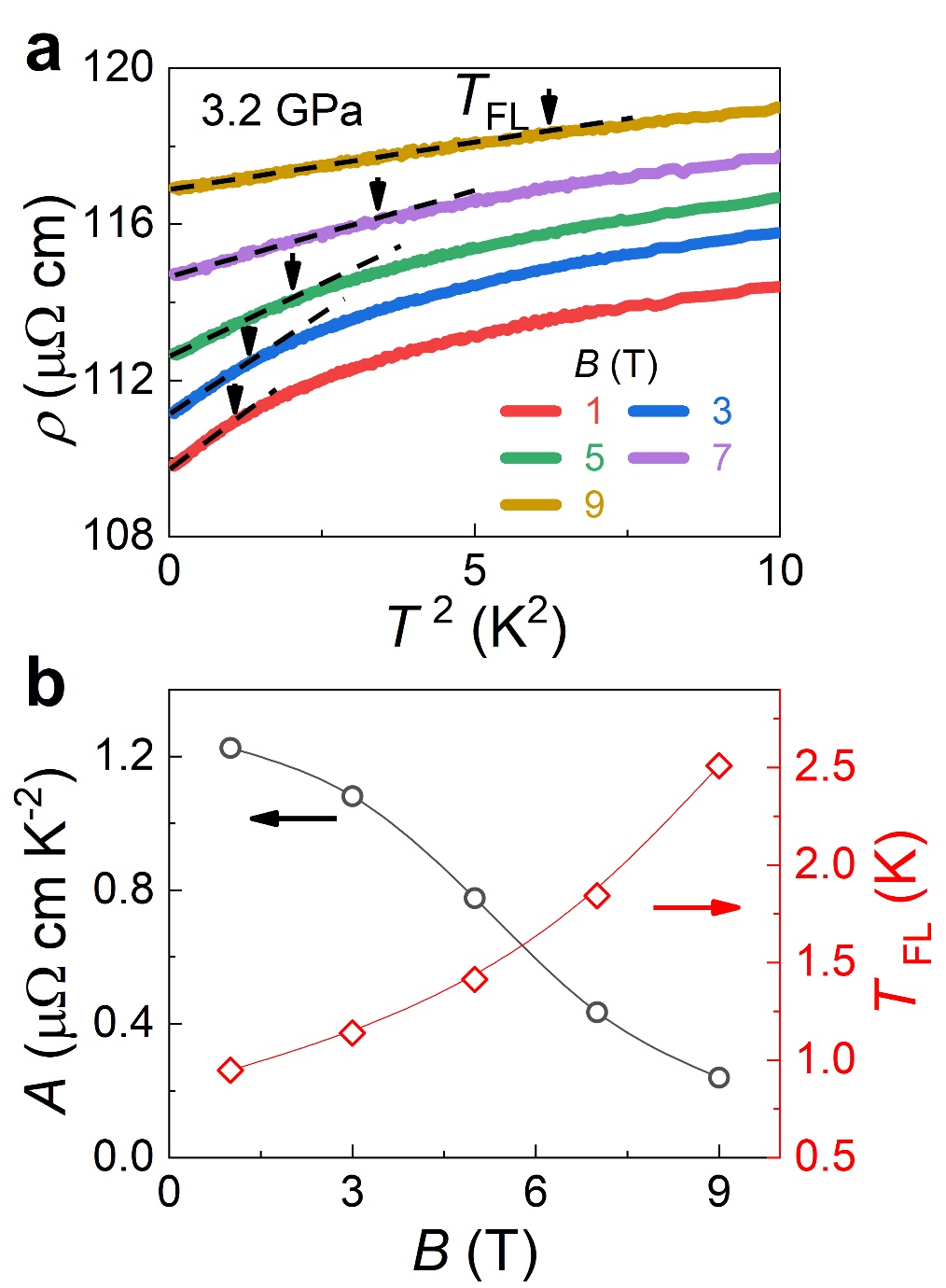


**Fig S7 | Evolution of Fermi-liquid behavior in Ce_5_CoGe_2_ under various magnetic fields at 3.2 GPa. a,** The black dashed lines show fits to a *T*^2^ dependence, corresponding to Fermi liquid behavior. The black arrows mark the Fermi-liquid temperature *T*_FL_ below which the resistivity follows the *T*^2^ behavior. All the curves are shifted vertically for clarity. **b,** Magnetic field dependence of the *A*-coefficient (black left axis) and the *T*_FL_ (red right axis).


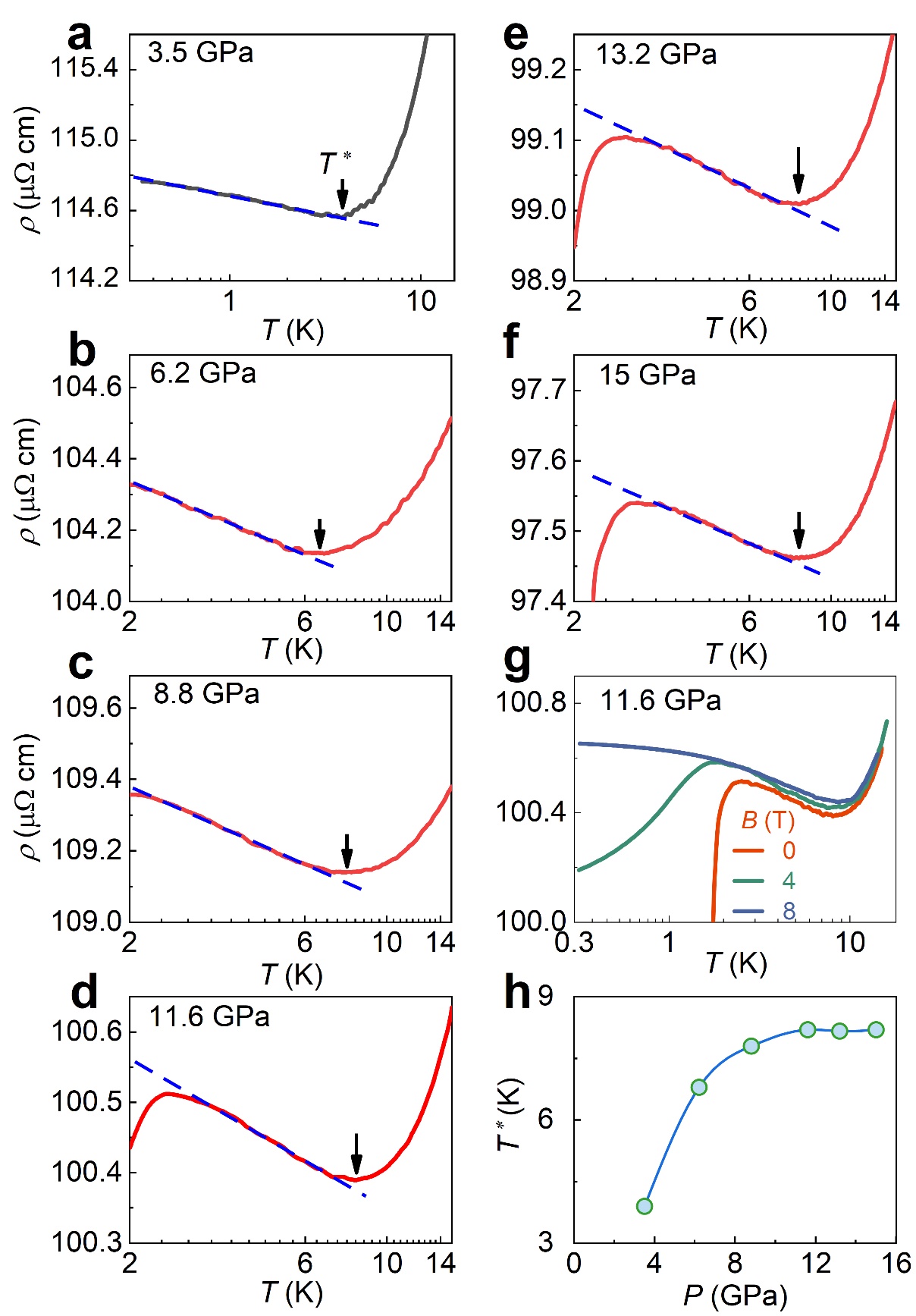


**Fig S8 | Low-temperature resistivity upturn under pressure. a–f**, *ρ*(*T*) at selected pressures from 3.5 to 15 GPa. The blue dashed lines represent a -log *T* fit. The black arrows indicate the temperature *T* ^*^, defined as the point below which the logarithmic behavior onsets. **g,** *ρ*(*T*) under various applied magnetic fields at 11.6 GPa. The superconducting transition is gradually suppressed with increasing field and fully suppressed at 8 T, while *T* * shows a negligible field dependence. **h**, Pressure dependence of *T* ^*^, showing a rapid increase at low pressures and saturation above 9 GPa.


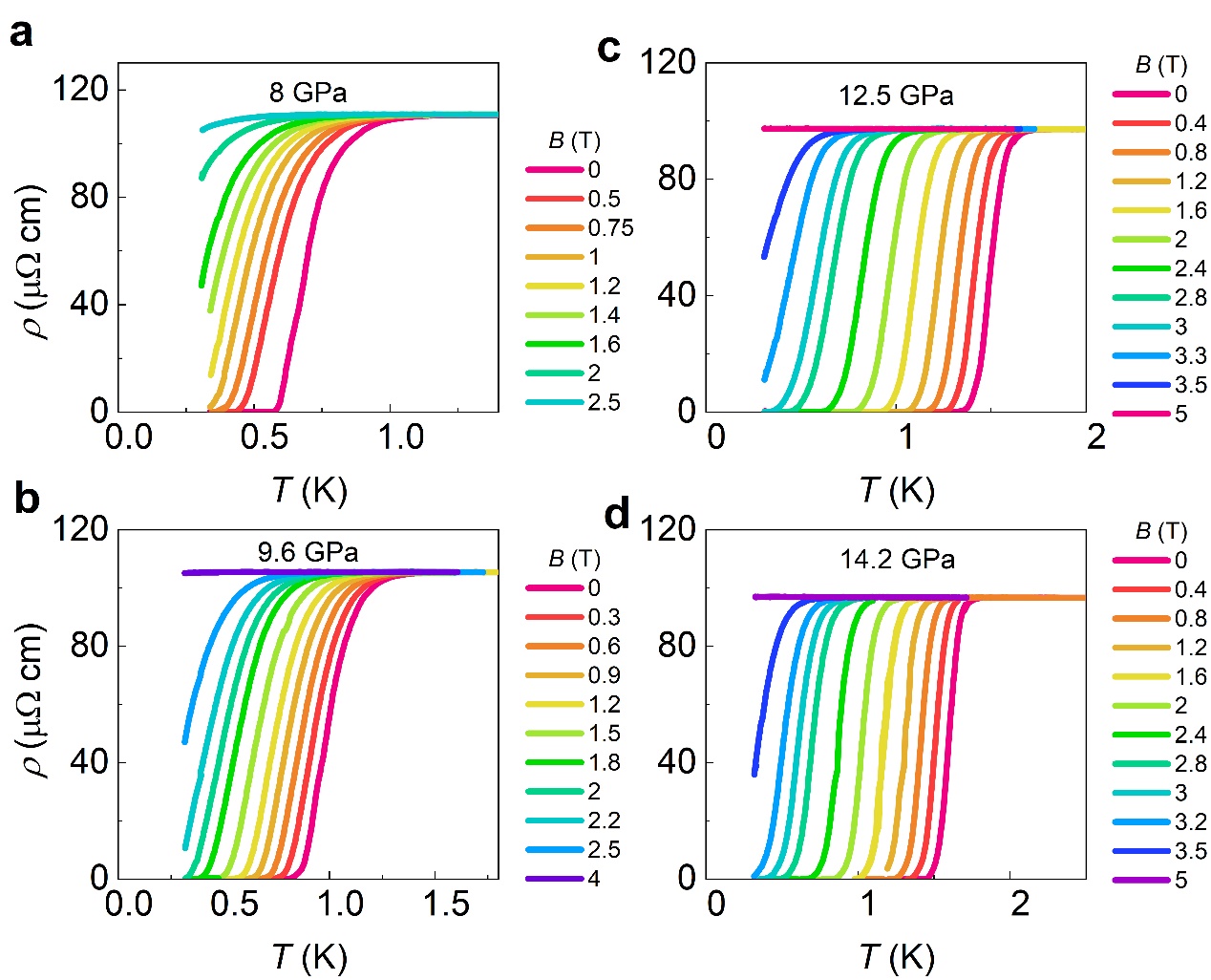


**Fig S9 | *ρ*(*T*) under various applied magnetic fields at selected pressures. a,** 8 GPa, **b,** 9.6 GPa, **c,** 12.8 GPa, **d**, 14.2 GPa.


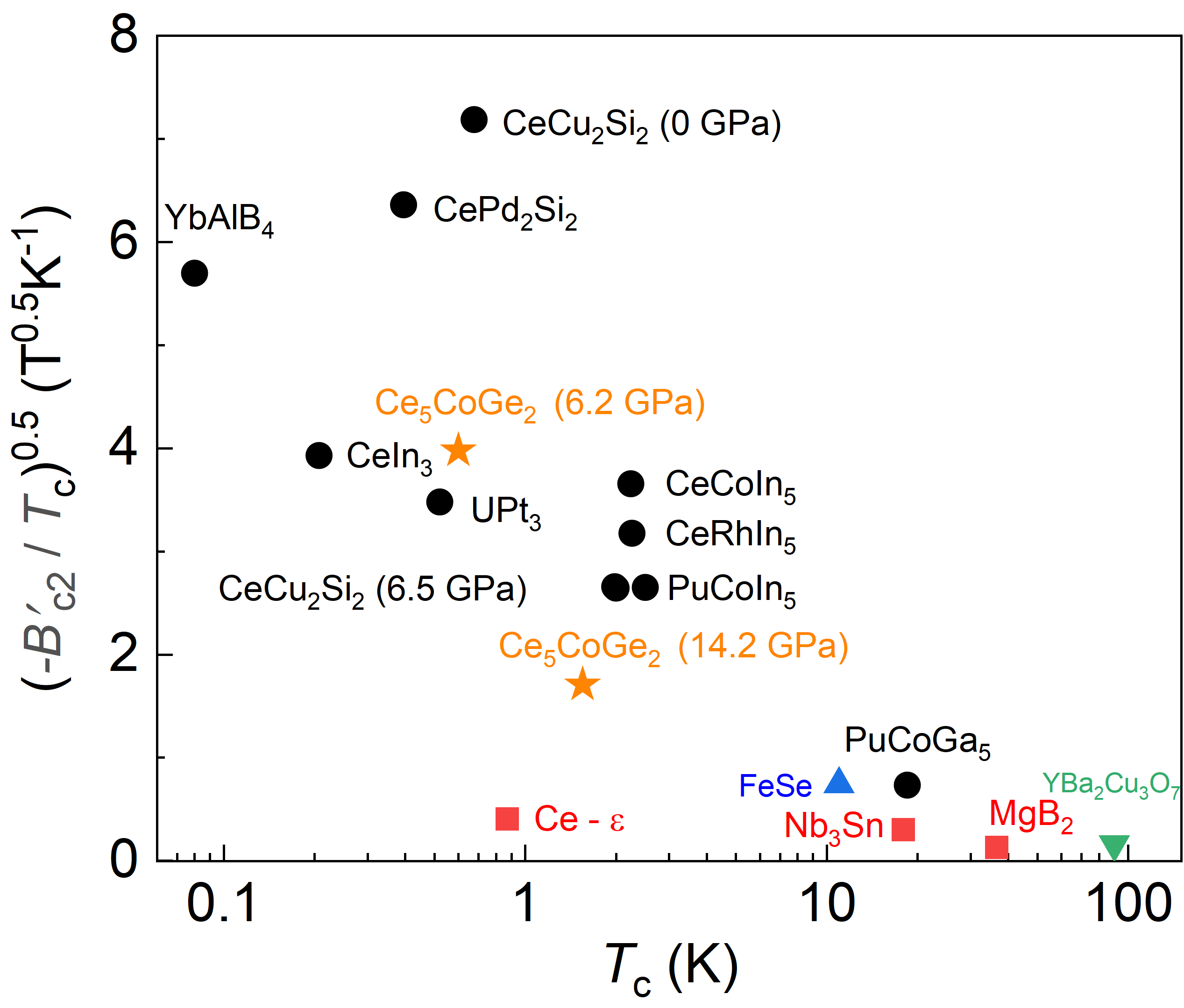


**Fig S10 | The variation of (*B*′_c2_/*T*_c_)^0.5^ with the superconducting transition temperature for various heavy-fermion compounds (circles)** ^[2–8]^**, Ce_5_CoGe_2_ (stars), iron-based (upward triangles)** ^[9,10]^**, cuprate (downward triangles)** ^[11]^**, and BCS superconductors (squares)** ^[12,13]^**, which qualitatively describe the differences in the effective carrier mass.**

[1] D. Su et al. *Phys. Rev. B* 110, 144432 (2024).

[2] U. Rauchschwalbe et al. *Phys. Rev. Lett.* 49, 1448 (1982).

[3] E. VargOZ et al. *Solid State Communications* 106, 631 (1998).

[4] E. D. Bauer et al. *J. Phys.: Condens. Matter* 24, 052206 (2011).

[5] K. Kuga et al. *Phys. Rev. Lett.* 101, 137004 (2008).

[6] G. Knebel et al. *Phys. Rev. B* 65, 024425 (2001).

[7] J. W. Chen et al. *Phys. Rev. B* 30, 1583 (1984).

[8] G. Knebel et al. *J. Phys. Soc. Jpn.* 77, 114704 (2008).

[9] S. I. Vedeneev et al. *Phys. Rev. B* 87, 134512 (2013).

[10] D. L. Sun et al. *Phys. Rev. B* 80, 144515 (2009).

[11] G. Grissonnanche et al. *Nat Commun* 5, 3280 (2014).

[12] I. N. Askerzade et al. *Supercond. Sci. Technol.* 15, L13 (2002).

[13] Y. N. Zhang et al. *Phys. Rev. B* 108, 094502 (2023).
